# Assessment of pre-referral treatment for malaria, diarrhea, and pneumonia by rural community health workers in Southwestern Uganda: a cross-sectional study

**DOI:** 10.1186/s12913-024-10598-9

**Published:** 2024-01-17

**Authors:** Michael Matte, Moses Ntaro, Jessica Kenney, Andrew Wesuta, Peter Chris Kawungezi, Shem Bwambale, David Ayebare, Stephen Baguma, Fred Bagenda, Geren Stone, Edgar Mulogo

**Affiliations:** 1 Department of Community Health, Mbarara University of Science and Technology, PO Box 1410, Mbarara, Uganda; 2 Center for Global Health, Massachusetts General Hospital, 125 Nashua Street, Boston, MA 02114 USA; 3 Bugoye Community Health Collaboration, Bugoye Health Centre III, PO Box 149, Kasese District, Uganda

**Keywords:** Community health workers, Integrated community case management, Pre-referral, Children under five, Rapid diagnostic test

## Abstract

**Background:**

Pre-referral treatment aims to stabilize the child’s condition before transferring them to a higher level of healthcare. This study explored pre-referral treatment for diarrhea, malaria and pneumonia in children U5. The study aims to assess pre-referral treatment practices among community health workers (CHWs) for children aged 2 to 59 months diagnosed with malaria, diarrhea, and pneumonia.

**Methods:**

Conducted in 2023, this study employed a quantitative retrospective analysis of secondary data gathered from March 2014 to December 2018. Among the subjects, 171 patients received pre-referral treatment, serving as the foundation for categorical data analysis, presenting proportions and 95% confidence intervals across different categories.

**Results:**

In this cohort, 90 (53%) of the 177 children U5 were male, and age distribution showed 39 (23%), 70 (41%), and 62 (36%) in the 2–11 months, 12–35 months, and 36–60 months categories, respectively. Rapid Diagnostic Test (RDT) malaria results indicated a negative outcome in 83(60%) and positive in 55 (40%) of cases. Symptomatically, 45 (26%) had diarrhea, 52 (30%) exhibited fast breathing, and 109 (63%) presented with fever. Furthermore, 59 (35%) displayed danger signs, while 104 (61%) sought medical attention within 24 h.

**Conclusion:**

The study analyzed a sample of 171 children under 5 years old to assess various characteristics and variables related to pre-referral treatment. The findings reveal notable proportions in gender distribution, age categories, RDT results, presence of diarrhea, fast breathing, fever, danger signs, and timely medical visits. The results highlight the need to strengthen pre-referral treatment interventions and enhance iCCM programs.

## Background

The burden of childhood illnesses in sub-Saharan Africa remains significant, with malaria, diarrhea, and pneumonia being leading causes of child morbidity and mortality, contributing nearly half of all deaths among children under the age of 5 years, especially in low-resource settings [1]. Pre-referral treatment aims to stabilize the child’s condition before transferring them to a higher level of healthcare, such as a health center or hospital, for more advanced care [2, 3]. It is an essential component of efforts to reduce child mortality and improve child health [4].Timely access to appropriate healthcare services is essential to reduce the burden of these illnesses and prevent associated deaths [5, 6].

The World Health Organization (WHO) and the United Nations Children’s Fund (UNICEF) have played critical roles in the development and promotion of integrated community case management (iCCM) initiatives for the treatment of common childhood illnesses in underserved communities [7]. These initiatives, including pre-referral treatment, aim to improve access to essential healthcare services for children under five years of age, particularly in areas with limited access to healthcare facilities [7–9].

Failure to provide pre-referral treatment, as evidenced in studies by Hetzel et al. (2023), is associated with prolonged illness and a higher risk of severe outcomes, resulting in increased morbidity and mortality among children under five, underscoring the critical importance of addressing this issue to mitigate adverse health consequences and enhance the effectiveness of integrated community case management programs [10–12].

Community health workers (CHWs) are trained healthcare providers operating within local communities to deliver vital healthcare services, education, and pre-referral treatments. They employ tools such as Rapid Diagnostic Tests (RDTs) for malaria, oral rehydration salts (ORS) for diarrhea management, respiratory rate timers for pneumonia assessment, and administer appropriate treatments, all while offering health education to the communities they serve [13, 14]. This comprehensive approach contributes to the well-being of the local population, encompassing pre-referral treatment for critical cases [11].

In certain scenarios, there is a risk of misunderstanding pre-referral medication, with caregivers mistakenly considering it as the conclusive treatment for children below the age of five. Emphasizing to caregivers that pre-referral medication is not the final treatment can greatly reinforce its purpose, even in less severe cases [15].

Community Health Workers (CHWs) are integral to Uganda’s integrated community case management (iCCM) strategy, endorsed by WHO and UNICEF, to address high mortality and morbidity rates in malaria, diarrhea, and pneumonia. CHWs receive essential medications, including ORS, RDTs malaria kits, Zinc, Amoxicillin, and timers through the Bugoye iCCM project, and they play a key role in assessing and treating children under five for these diseases, referring severe cases, providing pre-referral treatment, and establishing crucial links to healthcare facilities, thus contributing to the reduction of disease-related mortality and morbidity [11, 16].

According to the 2010 Uganda Ministry of Health Integrated Community Case Management (iCCM) guidelines, pre-referral treatment for children under the age of five is initiated when a child presents with specific symptoms [14, 17]. If a child has experienced fever for seven days or more, they are administered the initial dose of oral Anti-malarial ACT. In the presence of fever along with general danger signs like convulsions, Rectal Artesunate is administered [14, 17, 18].

Additionally, for cases involving chest indrawing or fast breathing, the first dose of amoxicillin is given, and if a child has persistent diarrhea for more than 14 days, the guidelines recommend commencing oral rehydration salt therapy [18]. These interventions are followed by the prompt referral of the child to the nearest healthcare facility for further medical support.

Pre-referral treatment administered by CHWs has emerged as a valuable strategy to bridge the gap between communities and formal healthcare facilities, ensuring prompt care for children under five with danger signs or severe illnesses. However, the success of pre-referral treatment depends on several factors, including community perceptions, the reliability of CHWs, and their ability to accurately identify and manage severe cases [19].

In rural South Western Uganda, where communities often face challenges related to geographical access, socio-economic factors, and healthcare infrastructure, Community Health Workers (CHWs) are at the forefront of providing pre-referral treatment for childhood illnesses [20, 21]. Comprehending the background and clinical characteristics of children under five who receive pre-referral treatment is crucial for optimizing community-based healthcare efforts. The study aims to assess pre-referral treatment practices among community health workers (CHWs) for children aged 2 to 59 months diagnosed with malaria, diarrhea, and pneumonia.

## Methods

### Study setting

Bugoye Sub-county is located in the Kasese District of Western Uganda. The Sub-county has a population of approximately 46,124 residents and 7,650 households distributed within 35 villages as of December 2019 [22]. The population of children below five years of age is 9,225 (20%). More than half of the Sub-county is mountainous and considerably hard to reach with a majority of low income subsistence residents [22]. 

CHWs are volunteers selected by their communities, trained for one week in iCCM based on Uganda Ministry of Health guidelines [23]. After the trainings, the CHWs are equipped with tools such as Sick child job aid, clinical and non-clinical supplies, iCCM CHW registers which they use to document and submit monthly data to Bugoye Community Health Collaboration (BCHC).

These iCCM CHWs registers for the period of four years and eight months were reviewed by the study team in mid-2022 to identify children under five years of age (2 to 59 months) who had been referred and offered pre-referral treatment.

### Study design

Study data was reviewed retrospectively by the program team for the study data starting March 2014 to December 2018.

### Data collection

One hundred and seventy-one (171) children U5 received pre-referral treatment and were thus included in the analysis. The 2010 Uganda Ministry of Health’s iCCM guidelines advise administering oral Anti-malarial ACT for children with fever lasting seven days or more and Rectal Artesunate for those with fever and general danger signs. In cases of chest indrawing or fast breathing, the first dose of amoxicillin is recommended, and prolonged diarrhea warrants the initiation of oral rehydration salt therapy [23]. All such cases are offered pre-referral treatment before being referred to a health facility.

A CHW assesses a child with a “sick child job aid” which helps to evaluate the state of the child as far as malaria, diarrhea and fast breathing [23]. A child who presents with danger signs of convulsions, vomiting, not breast feeding, chest in drawing and unconsciousness qualify for pre-referral treatment [23].

Data from the monthly cases malaria, diarrhea and pneumonia of children under five through the iCCM reporting forms were aggregated and entered into epi data software and extracted through excel for the period of March 2014 to December 2018. The data consisted of child’s age, village, disease condition diagnosed, medication provided, referral checkbox and follow-up checkbox. This data was cleaned, validated for inconsistencies and completeness. The CHWs record basic demographic information, diagnostic and observation results using the register.

### Data analysis

Data was exported from excel to STATA 12 for analysis. Descriptive statistics, including frequencies and percentages were used to present the proportion of pre-referral. The analysis included 171 cases to determine various characteristics and their proportions with corresponding 95% confidence intervals (CIs). Categorical variables such as gender, age categories, Rapid diagnostic test malaria results, presence of diarrhea, fast breathing, fever, danger signs, and medical attention-seeking within 24 h were examined. Frequencies and percentages were calculated for each variable category. Proportions with 95% CIs were generated to estimate the prevalence or distribution of each characteristic within the entire cohort.

## Results

### Background demographics and clinical characteristics and the magnitude of children under five years of age in the study

In this study, CHWs documented 171 children under 5 years old as candidates for pre-referral treatment and hence were included in the analysis. Among the group, 90 (53%) were males. Age distribution revealed 39 (23%) in the 2–11 months bracket, 70 (41%) in the 12–35 months range, and 62 (36%) aged 36–60 months. Rapid Diagnostic Test (RDT) malaria outcomes showed a negative result in 83 (60%) of cases and positive in 55 (40%). Symptomatically, 45 (26%) exhibited diarrhea, 52 (30%) had fast breathing, and 109 (63%) presented with fever. Additionally, 59 (35%) displayed danger signs, while 104 (61%) sought medical attention within 24 h. The characteristics are shown in Table 1.

**Table 1:** Background, clinical characteristics and proportions of pre-referral children under-fives

Characteristic/variables	All *N* = 171 n (%)	95% Confidence Interval
**Gender**		
Male Female	90 (53) 80 (47)	0.45–0.60 0.39–0.54
**Age category**		
2–11 months 12–35 months 36–60 months **Mean** = 24.2 months (SD = 16)	39 (23) 70 (41) 62(36)	0.17–0.30 0.34–0.49 0.28–0.42
**RDT results**		
Negative Positive	83 (60) 55 (40)	0.51–0.68 0.32–0.48
**Diarrhea**		
No Yes	126 (74) 45 (26)	0.67–0.80 0.20–0.33
**Fast breathing**		
No Yes	119 (70) 52 (30)	0.62–0.76 0.24–0.38
**Fever**		
No Yes	62 (36) 109 (63)	0.29–0.43 0.56–0.71
**Danger signs**		
No Yes	112 (66) 59 (35)	0.58–0.72 0.27–0.42
**Visit within 24 h**		
No Yes	67(39) 104 (61)	0.32–0.46 0.53–0.68

SD = Standard deviation

## Discussions

The study’s demographic data revealed that among the 171 children under observation, 53% were males and 47% were females. The age distribution exhibited a wide range, with 23% aged between 2 and 11 months, 41% between 12 and 35 months, and 36% between 36 and 60 months. This distribution across age groups suggests a comprehensive representation of young children within the study.

The study indicates that in general, a small proportion of children under 5 years of age received pre-referral treatment during the years of implementation period. Pre-referral treatment is a requirement for children who present with a danger sign to the CHWs [19]. As with iCCM programs elsewhere, the proportions of pre-referral treatments rates is low [10, 24]. The low pre-referral treatment rates could probably be because the number of children with danger signs that are seen by the CHWs is also low. Besides, the iCCM manual of the Uganda Ministry of Health lacks a defined expected range for pre-referral cases within iCCM, posing challenges for health monitoring and planning. It is possible that caregivers of children with danger signs go directly to the health facilities as the first point of care due to the anxiety around danger signs [16, 25, 26].

Furthermore, the Rapid Diagnostic Test (RDT) for malaria outcomes showed that 60% of cases had negative results, while 40% had positive results. This distribution is significant as it reflects a considerable portion of cases with positive RDT malaria outcomes, potentially indicating prevalent infections or susceptibility to certain conditions among the observed population. Studies carried out in various Africa countries showed that a significant number of children under-five with fever condition were given rectal artesunate as a pre-referral treatment before referral [24, 27]. Also, global trends for malaria, diarrhea and pneumonia have remained expressively high and more cases manifest in sub-Saharan Africa calling for pre-referral treatment among children [28]. Children with a positive malaria RDT result are treated with artemisinin-based combination therapy (ACT) or given rectal artesunate as pre-referral treatment before they’re referred to a higher facility level. This is consistent with the iCCM guidelines.

Another noteworthy finding is that around 61% of the cases sought medical attention within 24 h, indicating a positive trend in caregivers or parents’ prompt healthcare-seeking behavior. This behavior potentially plays a crucial role in facilitating early interventions and treatments and further credits the often less recognized efforts of CHWs in some rural settings.

### Limitation

The CHW register does not disaggregate the condition for which pre-referral treatment was given. It highlights whether or not pre-referral treatment was given. Nonetheless, the register provides information on the illness for which the child was evaluated by the CHW and whether or not diagnosis was performed.

Additionally, cross sectional study design was used and therefore, temporal relationships between factors and outcomes could not be established.

The data collection tool recommended for use by the CHWs does not record days since disease on set for malaria, diarrhea and pneumonia. The results therefore are limited by that factor. The retrospective design nature of this study is also another limitation since data quality relies on accuracy and completeness of the records.

The dataset comprising 171 children under five significantly reduced the sample size, limiting the scope for comprehensive inference from the results.

### Strength

Our study has several strengths. To the best of our knowledge, there are few studies addressing pre-referral treatment practices in routine iCCM among CHWs. Moreover, our study population is likely a representative sample of children seeking assistance from VHTs in the region.

## Conclusions

The study examined 171 children under the age of 5 to assess diverse characteristics and variables associated with pre-referral treatment. The outcomes reveal notable proportions in gender distribution, age categories, RDT results, the occurrence of diarrhea, fast breathing, fever, danger signs, and timely medical visits. These findings offer insights into the pre-referral treatment practices across distinct circumstances, emphasizing on focused interventions and enhanced strategies to boost the efficacy of (iCCM) programs in rural Uganda. The results highlight the need to strengthen pre-referral treatment interventions and enhance iCCM programs.

## Data Availability

Data are available upon reasonable request. This data are available for non-commercial use from the corresponding author (mattemichael18@gmail.com) upon reasonable request.

## Abbreviations

ACT: Artemisinin-based combination therapy
CHW: Community health worker
CI: Confidence interval
iCCM: Integrated community case management
RDT: Rapid Diagnostic Test for malaria
WHO: World Health Organization
